# Descriptive Representation and Reproductive Health Outcomes: The Role of Women Candidates in Reducing Teen Birth Rates

**DOI:** 10.3390/healthcare12202066

**Published:** 2024-10-17

**Authors:** Jeronimo Cortina, Shana Hardin

**Affiliations:** Department of Political Science and Population Health, University of Houston, Houston, TX 77204, USA; shardin4@cougarnet.uh.edu

**Keywords:** teen birth rates, health outcomes, women’s representation, social determinants of health

## Abstract

Background/Objectives: Teen birth rates remain a pressing public health issue in the United States, with significant long-term health risks for both mothers and children. Previous research has primarily focused on the impact of women in elected office on reproductive health outcomes, but less is known about the influence of women candidates. This study explores the relationship between the number of women candidates in county-level elections and county-level teen birth rates, highlighting the role of women candidates as visible role models and agents of potential policy change. Methods: We utilized a dataset covering county-level elections from 2010 to 2020, along with teen birth rate data from the CDC. We employed a multilevel model to analyze the relationship between the cumulative number of women candidates and teen birth rates, controlling for socioeconomic and health-related factors, such as insurance coverage and social vulnerability. Results: The findings indicate that as the number of women candidates increased, the estimated county-level teen birth rate declined. This effect was observed across different election years, with more substantial impacts in midterm and presidential elections. The analysis also confirmed that socioeconomic conditions, such as a lack of health insurance, contribute to higher teen birth rates. Conclusions: Women candidates, even when not elected, serve as important role models and influence reproductive health outcomes in their communities. Increasing the number of women candidates at the local level may be an effective strategy for reducing teen birth rates and improving public health outcomes.

## 1. Introduction

In 2023, Texas experienced its first increase in teen birth rates in 15 years, which brought renewed attention to reproductive health outcomes across the state [1]. While state-level trends are concerning, examining these rates at the county level reveals important local variations. Local factors, driven by community-specific policies and leadership, often play a more direct role in shaping reproductive health outcomes. One potential influence is the growing number of women candidates in local elections, whose presence can offer critical opportunities to address these challenges through targeted, community-driven approaches. Abortion bans and other health policies shape reproductive health outcomes, but the visibility of women candidates can signal additional aspirational beliefs and may influence outcomes for young women. Descriptive representation, where individuals see candidates or leaders who reflect their characteristics, allows young girls to imagine themselves in positions of influence and ambition. Research shows that simply seeing women running for office can positively shape the aspirations and behaviors of adolescent girls, even if these women do not win. Initial studies explore how the presence of women in politics can reduce adverse health outcomes for young women [2,3].

Descriptive representation has been widely studied for its influence on various social outcomes, primarily through role model effects. When leaders or candidates share traits or experiences with their constituents, it can affect both policy priorities and the aspirations of those represented [4]. Research in political science consistently shows that in contexts where women are more visible in politics, adolescent girls are more likely to engage in political and policy discussions and express leadership ambitions [5]. The presence of visible and viable women candidates has been associated with an increase in adolescent girls’ anticipated civic participation [6]. This body of work suggests that high-profile women in politics serve as role models, inspiring other women and girls to pursue careers in politics and public policy, thus increasing the overall number of women candidates [7]. The novelty of women candidates can catalyze increased discussions about politics and public policy issues among younger women, further fostering civic engagement [8].

The role of women in leadership extends beyond politics into challenging traditional gender roles and raising aspirations. Research has demonstrated that young girls in areas with women leaders tend to spend less time on domestic chores and aspire to broader career opportunities outside the home [9]. These findings suggest that the aspirations of young girls are shaped by exposure to women in leadership, which alters their perceptions of societal roles and can lead to more positive outcomes. These findings are integral to understanding how descriptive representation can influence behavior and expectations in the political sphere and across broader social contexts.

Extant research on role model effects also highlights their impact on health behaviors. For example, studies show that African American teachers reduce teen pregnancy rates among African American girls by serving as role models [2]. Similarly, women candidates at the local level may draw attention to public health and reproductive health services, contributing to reductions in teen birth rates. While much of the existing research focuses on women in elected office, emerging evidence suggests that the presence of women candidates alone can signal potential shifts in reproductive healthcare policy, including contraception and prenatal care, which are issues women in leadership positions often prioritize [3,10].

Research on community-based role model interventions further demonstrates the complexity of influencing health behaviors. Programs targeting adolescent girls—such as girl groups—have shown potential for improving attitudes toward gender roles, early pregnancy, and child marriage; however, their impact on health outcomes has been mixed [11]. These studies highlight the challenges of relying solely on local interventions to improve reproductive health outcomes. Increasing women’s representation in local government could strengthen these programs by fostering community trust and reinforcing public health priorities. Evidence suggests that women in elected office are associated with improved health outcomes, including reductions in maternal and child mortality and increased life expectancy [12,13,14]. Additionally, women mayors tend to allocate more resources to healthcare, particularly on issues affecting women and girls [15]. While the direct role of elected women in shaping policy is well-established, this study explores the broader role of women candidates in influencing public health outcomes. The presence of women candidates at the county level, even those not elected, may foster aspirational beliefs among young women and contribute to strengthening public health efforts, particularly in the realm of reproductive health.

The impact that women candidates may have on health outcomes, particularly reproductive health outcomes, is likely to be more pronounced at the local level. County officials are often more closely connected to their communities than their state or national counterparts, enabling them to respond to local needs and implement tailored public health campaigns that resonate with at-risk populations [10]. Women candidates running for county positions could send a strong signal of change, even if they do not ultimately win their races. Their candidacies can foster community discussions around health issues like teen pregnancy and signal the importance of reproductive health services to the local population. Additionally, existing research finds that attainable role models, such as local candidates, inspire young women more effectively than those in positions often deemed unattainable, like the presidency or other higher offices [16]. This makes local candidates particularly important role models, given their proximity and accessibility to the community. Counties with more women candidates may see greater investment in reproductive health and stronger partnerships with schools, nonprofits, and local health agencies [3]. The visibility of these candidates creates an environment where young women may feel inspired to pursue opportunities beyond early pregnancy [17].

The current study explores the impact of an increase in the number of women candidates in county-level elections on teen birth rates. Using candidate data from a comprehensive database on local elections [18] and teen birth rate data from the CDC, we analyzed trends from 2010 to 2020 across U.S. counties. Our results suggested that as the cumulative number of women candidates increased, the estimated county-level teen birth rate for the 15–19 age group decreased. This relationship underscores the importance of women candidates as visible figures who inspire young girls to pursue broader opportunities and make healthier decisions, supporting the role model theory of descriptive representation.

The urgency of addressing teen birth rates is underscored by recent research showing that having a child as a teenager can significantly increase the risk of premature mortality among women and girls [19]. By examining how women leaders and candidates impact reproductive health outcomes, this research highlights the importance of having women in office and also striving for political positions. The findings have significant policy implications, suggesting that increasing the number of women candidates and women in local offices at the county level may be an essential strategy for reducing teen birth rates and improving reproductive health outcomes for young women.

## 2. Materials and Methods

The data utilized in this study came from three primary sources: women candidate data came from the American Local Government Elections Database (ALGED), a new database covering local government elections in counties across the U.S. [18]; teen birth rate data from the CDC [20]; and additional supplemental variables from PolicyMap, which provides curated and normalized data from various sources [21]. The dependent variable was the estimated county-level teen birth rate for the 15–19 age group (per 1000 females aged 15–19) from 2010 to 2020, as reported by the CDC National Center for Health Statistics. The primary predictor was the cumulative number of women candidates across 944 counties during this period when data are available. We selected this variable to illustrate the cumulative effects of descriptive representation on potential policy changes and the sustained impact as role models. Policy changes are rarely implemented immediately [22], and even when they are, their outcomes are typically not felt in the short term but only after some time.

Based on current literature, we also controlled for the most salient electoral and ecological variables to examine the association between the cumulative number of women candidates and county-level teen birth rates. First, we included whether the candidate won in a given year to control for the electoral success of women being elected, their incumbency status due to the electoral advantage it confers [23], and whether the candidate belonged to a racial minority group to control for the likelihood of their electoral success. Recent research shows that minority women candidates have had growing success in local elections [24], the vote share they received accounting for the margin of victory and the type of office they ran for, including county executive, county legislator, prosecutor, or sheriff, controlling for the impact that different types of elections may have on the electorate [25].

Additionally, we included county-level estimates of the percentage of women aged 16–19 who were not enrolled in school and were unemployed or not in the labor force to account for the impact of high school dropout rates and school disengagement on the likelihood of adolescent pregnancy [26]. We also accounted for the estimated percentage change in the uninsured population between 2012–2016 and 2017–2021. Having health insurance has been associated with a reduction in teen pregnancy rates, primarily because it increases access to reproductive health services, including contraception and family planning [27], as well as the estimated percentage change in the population living in poverty over the same periods, which accounts for its impacts on limited access to education, healthcare, and contraception, all of which contribute to increased risks of teen pregnancies [2]. Lastly, we included the 2020 Social Vulnerability Index (SVI), which measures populations most in need of support during disasters or extreme weather events. The index consists of four categories of vulnerability: socioeconomic status, household composition and disability, minority status and language, and housing and transportation, all sourced from PolicyMap. The Social Vulnerability Index (SVI) holds significant importance, as it identifies areas with the greatest need for policy interventions [28]. Women candidates can play a crucial role in advocating for and implementing such interventions, as their leadership can help address the specific needs of vulnerable communities and highlight the importance of targeted policies.

### 2.1. Data Procedures

As a first step, local county-level data elections from ALGED [18], teen birth rate data from the National Center for Health Statistics [20], and additional data from PolicyMap [21] were merged using each county’s unique Federal Information Processing Standard (FIPS) code by year to construct a tabular, cross-sectional dataset among the 944 counties that had local elections (i.e., county executive, county legislator, prosecutor, and/or sheriff) during 2010–2020. The total county sample size (*n* = 944) represented 30% of all counties in the United States; however, not all counties in the United States run elections for the same office. For instance, the number of counties with elected sheriffs varies by state. Texas has the greatest number of sheriffs, with 251, while Alaska, Connecticut, Washington D.C., Hawaii, and Rhode Island have no elected sheriffs [29]. Data were recorded for ease of interpretation, as indicated in Table 1, which summarizes the data.

### 2.2. Statistical Analysis

To test the model, we estimated a multilevel random-effects linear regression model (see Equation (1)) to analyze how different individual and county-level factors impacted the teen birth rate while accounting for the hierarchical structure of the data (counties nested within states). This modeling strategy allowed for random variation at both the county and state levels, which meant it could account for unobserved heterogeneity that might affect the birth rate differently in different counties and states [30]. In other words, given the hierarchical data structure, random effects, compared to fixed effects, allowed for both individual and group-level variation, that is, within and between counties. Varying intercepts accounted for differences between counties, while varying slopes accounted for differences in how variables affected the outcome within each county. In this model strategy, each county had its own baseline level (random intercepts). Additionally, each slope (random slope) allowed the effect of our main predictor to vary across counties, capturing the impact where both the cumulative presence of women candidates and its effect differed by county.

From a substantive perspective, random effects are more appropriate than fixed effects to account for the different political and electoral dynamics at the county and state levels.

(1) $$Y_{\mathrm{ijt}}=\beta_{0}+\beta_{1}(X_{1\mathrm{ijt}})+\beta_{2}(X_{2\mathrm{ijt}})+\beta_{3}(X_{3\mathrm{ijt}})+\beta_{4}(X_{4\mathrm{ijt}})+\beta_{5}(X_{5\mathrm{ijt}})+\beta_{6}(X_{6\mathrm{ijt}})+\beta_{7}(X_{7\mathrm{ijt}})+\beta_{8}(X_{8\mathrm{ijt}})+\beta_{9}(X_{9\mathrm{ijt}})+\beta_{10}(X_{10\mathrm{ijt}})+\beta_{11}(X_{11\mathrm{ijt}})+\;u_{j}+v_{k}+\varepsilon_{\mathrm{ijt}}$$

where:

- Y: Dependent variable representing the teen birth rate for counties.
- X_1_: Continuous variable representing the gender estimation for candidates.
- X_2_: Binary variable indicating whether the candidate won the election.
- X_3_: Binary variable indicating if the candidate is the incumbent.
- X_4_: Political identification of the candidate.
- X_5_: Candidate’s race.
- X_6_: The vote share received by the candidate.
- X_7_: Categorical variable for the type of office (county executive, legislator, etc.).
- X_8_: Estimated percentage of females aged 16–19 not in school or the labor force.
- X_9_**:** Percentage change in the population without health insurance.
- X_10_: Percentage change in the population living in poverty.
- X_11_: Social vulnerability index or other contextual measures.

Random effects:

- u_j_: Random effect for county.
- v_k_: Random effect for state.
- ε_ijt_: Residual error term for individual observations.

To account for the differences between years, we estimated Equation 1 by election year, that is, county elections that occurred during odd years (*n* = 4060 county/year election candidates), midterm election years (*n* = 8932 county/year election candidates), and presidential election years (*n* = 8300 county/year election candidates) [25].

## 3. Results

Table 2 presents the results after estimating a series of multilevel random-effects linear regression models by election year.

Overall, the results suggest that as the cumulative number of women candidates increased, the estimated county-level teen birth rate for the 15–19 age group (per 1000 females aged 15–19) decreased. In odd election years, an increase of one in the cumulative number of women candidates corresponded to a decrease of 0.25 in the estimated teen birth rate (*p* < 0.05), holding everything else constant. During midterm election years, the teen birth rate declined by 0.46 (*p* < 0.01), while in presidential election years, the rate decreased by 0.40 (*p* < 0.01).

Regarding the control variables, as the percentage of people without health insurance increased, the county-level teen birth rate for the 15–19 age group rose by 0.06 (*p* < 0.01) in midterm elections and by 0.08 (*p* < 0.01) in presidential election years. Consistent with existing literature [28], an increase in the county-level social vulnerability index correlated with a rise in teen birth rates.

The random effects demonstrated significant variation, likely due to differences in health policies and healthcare infrastructure at the county and state levels. This variation underscores the importance of such a modeling strategy when understanding teen birth rates. The Wald chi-square test evaluated the null hypothesis that the fixed effects were simultaneously zero. For each of the models, the Wald χ^2^ test, with 13 degrees of freedom and *p*-values of zero, suggested that the model’s predictors significantly explained the variability in teen birth rates. Additionally, the LR test compared the random effects model against a linear model without random effects. In each case, the LR test yielded an χ^2^ with *p*-values of zero, indicating that the model provides a significantly better fit than a simpler linear model without random effects.

**Figure 1:**
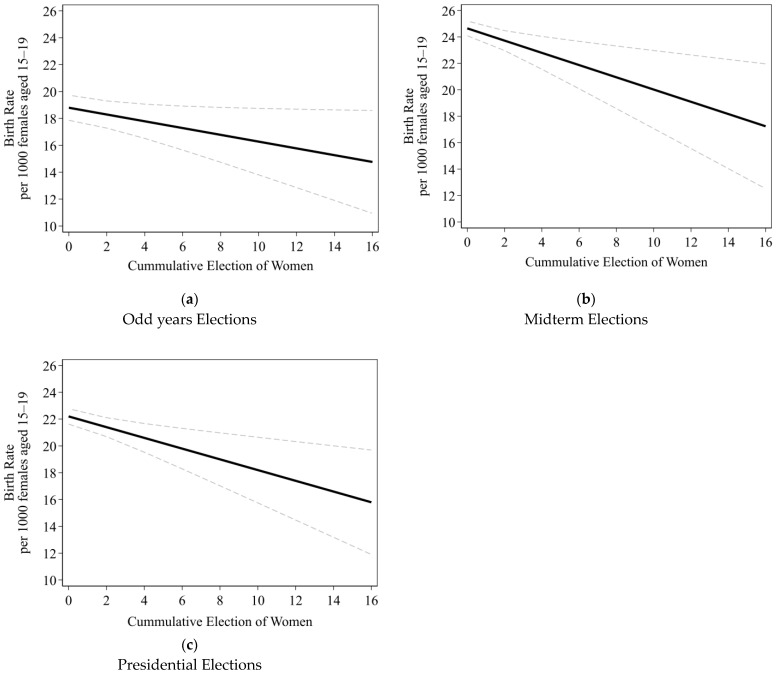
Multilevel random-effects linear regression models predicting the estimated county-level teen birth rate for the 15–19 age group (per 1000 females aged 15–19) (black solid line) with 95% confidence intervals (black dotted line), given the cumulative number of women running for office by (**a**) odd election years; (**b**) midterm elections years; and (**c**) presidential elections years.

Figure 1 presents the marginal effects of the cumulative number of women running for office on the estimated county-level teen birth rate for the 15–19 age group (per 1000 females aged 15–19) to illustrate the relationship between the cumulative number of women running for office and the estimated county-level teen birth rate. These figures highlight how increasing the number of women candidates influenced the reduction in teen birth rates at the county level.

Figure 1 illustrates that as the number of women candidates running for office increased, the estimated teen birth rate decreased. In odd-year elections, the estimated teen birth rate with no women candidates was about 18.79 births per 1000 females aged 15–19. As the cumulative number of women candidates rose to 16, the estimated teen birth rate decreased to 14.76 births per 1000 females aged 15–19, representing a 21.45% reduction. During midterm election years, the estimated birth rate decreased from 24.65 to 17.24 births per 1000 females aged 15–19, a 30.05% decrease. Similarly, in presidential election years, the rate dropped by 28.85%, from 22.20 to 15.79 births per 1000 females aged 15–19.

Overall, across different election years, we observed the same downward correlation between the number of women candidates and the teen pregnancy rate. However, the rate of reduction varied somewhat, depending on the type of election. These variations highlighted the differential impact of each election cycle [25]. A key difference lies in voter turnout. Presidential elections tend to have higher turnout than midterm and gubernatorial elections, while turnout in odd-year elections tends to be lower. This lower turnout emphasizes the localized nature of those elections and their candidates, emphasizing the local connection as well as being a more attainable position.

## 4. Discussion

The findings from this study provide strong evidence that as the cumulative number of women candidates increases, teen birth rates at the county level decline. These results suggest the critical role that women candidates play in influencing reproductive health outcomes, even when they are not elected. While much of the existing research focuses on the health impacts of women in office [3,10,12,13,14,15], our study shows that the presence of women candidates alone can have significant effects on public health. These findings highlight a broader scope of influence that extends beyond traditional officeholders.

One key contribution of this study is the evidence that women candidates may act as visible role models, plausibly influencing health behaviors in ways other role models, such as teachers, cannot. Prior research has shown that African American teachers, for example, reduce teen pregnancies among African American girls [2]. However, teachers lack the direct political and policy-making influence that candidates wield. Women candidates, even those not elected, signal potential shifts in policy and serve as broader role models for young women, showing them that striving for leadership positions is attainable. This study expands the understanding of role model effects and highlights the importance of political visibility.

While this study demonstrates a significant correlation between the presence of women candidates and lower teen birth rates, the underlying mechanisms driving this relationship warrant further investigation. One possible explanation is the role of social signaling, where women candidates shift societal norms and expectations, encouraging young women to pursue educational and career opportunities that may reduce the likelihood of early pregnancy. Additionally, women candidates often advocate for policies that promote women’s health and access to reproductive services, which could indirectly affect teen birth rates. For instance, a potential indirect way would be via decreasing the SVI in their communities by advocating and implementing interventions that could address issues related to underserved populations, such as quality and availability of housing, language barriers, and transportation. Further research should explore how the platforms, policy priorities, and visibility of these candidates contribute to shaping health behaviors in their communities. Investigating whether these effects are mediated by local health services, community engagement, or public health campaigns initiated by women candidates could deepen our understanding of these mechanisms. Research should also examine whether these effects are consistent across different geographic regions or population groups to better understand the broader implications of women’s candidacies on public health.

The policy implications of these findings are significant. Increasing the number of women candidates at the local level may be an effective strategy for addressing teen pregnancy rates and broader reproductive health challenges. Policymakers and community leaders should consider initiatives encouraging more women to run for office, including training programs, mentorship opportunities, and resources that lower the barriers to candidacy for women, especially in underrepresented communities. Supporting the candidacies of women who advocate for reproductive health services, sex education, and comprehensive healthcare access could help amplify the public health benefits associated with women’s political participation. Local governments and advocacy organizations might also explore partnerships that connect women candidates with health-focused community initiatives, thereby reinforcing the impact of their candidacies on public health outcomes.

The current study also complements research showing that women in office improve overall health outcomes, such as life expectancy [12,13,14]. Our findings add to this body of work by demonstrating that the cumulative presence of women candidates is correlated with lower teen pregnancy rates, which can positively impact reproductive health. This work underscores the importance of descriptive representation and the broader influence women’s political participation can have on public health.

It is essential to recognize the broader context in which these findings matter. Teen pregnancy is not only a reproductive health issue but also one that carries long-term health risks [31], including premature mortality [19]. Moreover, socioeconomic conditions, including economic inequality, play a significant role in influencing pregnancy rates among teenagers [32]. Addressing teen pregnancy requires community-focused interventions that not only provide health services but also emphasize educational and career development for young women [33].

Teen births have significant consequences, not only for young mothers but also for their children. Research shows that children born to teenage mothers tend to have poorer educational, behavioral, and health outcomes [34]. Moreover, the U.S. continues to rank among higher-income countries with one of the highest teen birth rates [35,36], which highlights the pressing need for public health interventions to reduce these rates and improve outcomes for both mothers and children. Overall, teen pregnancies are associated with a range of negative health outcomes, including premature births, low birth weights, and neonatal death [37].

### Limitations

Working with observational data entails certain limitations. First, this study focuses on counties that held local elections (i.e., county executive, county legislator, prosecutor, and/or sheriff) between 2010 and 2020, which limits the generalizability of the findings to other counties with different political and healthcare structures, as well as to more extended time periods. Second, as with any candidate running for office, there is an element of self-selection; however, there is no credible mechanism to suggest an endogenous relationship between candidates choosing to run for office, whether man or woman, and teen birth rates at the county level. In addition, the data on women’s candidates may be skewed. That is, there may be some counties or states in which more women would be more likely to run for office than in other counties or states, which could impact the results of this paper. Nonetheless, our sample, which includes 944 counties and 48 states, is broad enough to account for such differences, which are incorporated into our modeling strategy. Third, while the study demonstrates a correlation between the increase in women candidates and a decrease in teen birth rates, it does not definitively establish causality, nor does it claim to do so. Instead, this study limits its conclusions to highlighting the correlation between these two variables while suggesting potential causal mechanisms, being careful not to commit the ecological fallacy—using aggregate data to infer individual-level behaviors. Fourth, although this study controls for several factors that have been associated with teen births in the current relevant literature, other variables may not be accounted for, due to the lack of reliable and valid data at the county level, such as access to sex education programs or the influence of religious and cultural norms that could affect teen birth rates.

These limitations suggest areas for further research and caution in interpreting the findings.

## 5. Conclusions

In conclusion, this study highlights the significance of women candidates in reducing teen birth rates and the broader potential role of political representation in shaping public health outcomes. By demonstrating that the influence of women extends beyond those who are elected, this research suggests that increasing the number of women candidates may be a key strategy in addressing critical public health issues like teen pregnancy.

## Figures and Tables

**Table 1:** Summary statistics.

Variable	Obs.	Mean	Std. dev.	Min	Max
Teen birth rate	21,292	21.85	12.05	1.50	93.70
Cumulative # of women candidates	21,292	0.30	0.59	0.00	17.00
Winner (1 = yes, 0 = no)	21,292	0.56	0.50	0.00	1.00
Incumbent (1 = yes, 0 = no)	21,292	0.42	0.98	0.00	31.00
Partisanship (1 = Dem, 0 = GOP)	21,292	0.50	0.50	0.00	1.00
Race (1 = minority, 0 = white)	21,292	0.16	0.36	0.00	1.00
% Vote Share	21,292	0.52	0.28	0.00	1.00
Office					
County Executive	21,292	0.03	0.18	0.00	1.00
County Legislator	21,292	0.72	0.45	0.00	1.00
Prosecutor	21,292	0.09	0.28	0.00	1.00
Sheriff	21,292	0.16	0.37	0.00	1.00
% Disconnected female youth	21,292	6.37	2.59	0.61	18.80
% People w/o health insurance	21,292	−25.88	13.60	−58.06	34.15
% People living in poverty	21,279	−268.09	499.83	−10,194.12	7450.00
Social vulnerability index	21,292	0.52	0.26	0.01	1.00

**Table 2:** Multilevel random-effects linear regression models by election year predicting teen birth rates at the county level.

	Odd Year	Midterm Years	Presidential Years
	Elections	Elections	Elections
Cumulative # of women running for office	−0.25 **	−0.46 ***	−0.40 ***
	(0.12)	(0.15)	(0.13)
Winner (1 = yes, 0 = no)	−0.37 **	0.30	0.09
	(0.17)	(0.21)	(0.19)
Incumbent (1 = yes, 0 = no)	0.03	−0.14	0.06
	(0.08)	(0.09)	(0.08)
Partisanship (1 = Dem, 0 = GOP)	0.12	−0.47 ***	0.10
	(0.13)	(0.16)	(0.15)
Race (1 = minority, 0 = white)	−0.17	−0.26	−1.00 ***
	(0.20)	(0.23)	(0.21)
% Vote Share	0.46	−0.91 **	−0.76 **
	(0.38)	(0.43)	(0.38)
Office			
County Legislator	−0.20	−1.32 ***	−0.08
	(0.31)	(0.47)	(0.57)
Prosecutor	0.68 *	2.14 ***	1.46 **
	(0.40)	(0.55)	(0.62)
Sheriff	0.33	−0.99 **	0.55
	(0.36)	(0.50)	(0.60)
% Disconnected female youth	2.17 ***	1.90 ***	2.04 ***
	(0.19)	(0.12)	(0.12)
% People w/o health insurance	0.01	0.06 ***	0.08 ***
	(0.03)	(0.02)	(0.02)
% People living in poverty	0.00	−0.00	−0.00 ***
	(0.00)	(0.00)	(0.00)
Social vulnerability index	14.98 ***	19.95 ***	15.49 ***
	(2.11)	(1.24)	(1.26)
Constant	−0.26	4.95 ***	2.23 **
	(1.50)	(1.00)	(1.08)
Var (County)	0.89	0.29	0.32
	(0.75)	(0.94)	(1.04)
Var (State)	1.79 ***	1.84 ***	1.88 ***
	(0.13)	(0.05)	(0.06)
Var (Residual)	1.36 ***	1.92 ***	1.78 ***
	(0.01)	(0.01)	(0.01)
Observations	4060	8931	8288
Number of groups	222	538	533
Wald χ^2^ (13)	465	3269	1124
LR Test vs. Linear Model (χ^2^ = 2)	3443	3443	3802

Standard errors in parentheses. *** *p* < 0.01, ** *p* < 0.05, * *p* < 0.1.

## Data Availability

Election-related data are available at https://osf.io/mv5e6/ (accessed on 6 September 2024), and teen pregnancy rates are available at https://www.cdc.gov/nchs/data-visualization/county-teen-births/index.htm (accessed on 6 September 2024).

## References

[1] Gamboa S. Texas Teen Birthrate Rose for First Time in 15 Years after Abortion Ban, Largely Affecting Latinas. https://www.nbcnews.com/news/latino/texas-latina-teen-birth-rate-rises-after-abortion-ban-rcna135511.

[2] Atkins D.N., Wilkins V.M. (2013). Going beyond reading, writing, and arithmetic: The effects of teacher representation on teen pregnancy rates. J. Public Adm. Res. Theory.

[3] Paxton P., Kunovich S., Hughes M.M. (2007). Gender in politics. Annu. Rev. Sociol..

[4] Pitkin H.F. (2023). The Concept of Representation.

[5] Wolbrecht C., Campbell D.E. (2007). Leading by example: Female members of parliament as political role models. Am. J. Polit. Sci..

[6] Campbell D.E., Wolbrecht C. (2006). See Jane run: Women politicians as role models for adolescents. J. Politics.

[7] Ladam C., Harden J.J., Windett J.H. (2018). Prominent role models: High-profile female politicians and the emergence of women as candidates for public office. Am. J. Polit. Sci..

[8] Wolbrecht C., Campbell D.E. (2017). Role models revisited: Youth, novelty, and the impact of female candidates. Politics Groups Identities.

[9] Beaman L., Duflo E., Pande R., Topalova P. (2012). Female leadership raises aspirations and educational attainment for girls: A policy experiment in India. Science.

[10] Bhalotra S., Clots-Figueras I. (2014). Health and the political agency of women. Am. Econ. J. Econ. Policy.

[11] Temin M., Heck C.J. (2020). Close to home: Evidence on the impact of community-based girl groups. Glob. Health Sci. Pract..

[12] Shen C., Williamson J.B. (1997). Child mortality, women’s status, economic dependency, and state strength: A cross-national study of less developed countries. Soc. Forces.

[13] Hessel P., González Jaramillo M.J., Rasella D., Duran A.C., Sarmiento O.L. (2020). Increases in Women’s Political Representation Associated with Reductions in Child Mortality in Brazil: Study assesses the effects of female political representation on mortality among children younger than age five in Brazil. Health Aff..

[14] Ng E., Muntaner C. (2018). The effect of women in government on population health: An ecological analysis among Canadian provinces, 1976–2009. SSM-Popul. Health.

[15] Funk K.D., Philips A.Q. (2019). Representative budgeting: Women mayors and the composition of spending in local governments. Political Res. Q..

[16] Schneider M.C., Sweet-Cushman J., Gordon T. (2023). Role Model Do No HARM: Modeling Achievable Success Inspires Social Belonging and Women’s Candidate Emergence. J. Women Politics Policy.

[17] Pell B., Hawkins J., Cannings-John R., Charles J.M., Hallingberg B., Moore G., Roberts J., Van Sluijs E., Morgan K. (2022). CHoosing Active Role Models to INspire Girls (CHARMING): Protocol for a cluster randomised feasibility trial of a school-based, community-linked programme to increase physical activity levels in 9–10-year-old girls. Pilot Feasibility Stud..

[18] de Benedictis-Kessner J., Lee D.D.I., Velez Y.R., Warshaw C. (2023). American local government elections database. Sci. Data.

[19] Ray J.G., Fu L., Austin P.C., Park A.L., Brown H.K., Grandi S.M., Vandermorris A., Boblitz A., Cohen E. (2024). Teen pregnancy and risk of premature mortality. JAMA Netw. Open.

[20] Khan D., Hamilton B., Rossen L., He Y., Wei R., Dienes E., National Center for Health Statistics (2022). Teen Birth Rates for Age Group 15–19 in the United States by County, 2003–2020. https://www.cdc.gov/nchs/data-visualization/county-teen-births/index.htm.

[21] PolicyMap (2024). PolicyMap. Philadelphia, PA. https://www.policymap.com/.

[22] True J., Jones B., Baumgartner F. (2007). Explaining stability and change in public policymaking. Theories of the Policy Process.

[23] Gelman A., King G. (1990). Estimating incumbency advantage without bias. Am. J. Polit. Sci..

[24] Perry A.M. (2018). Analysis of Black Women’s Electoral Strength in an Era of Fractured Politics.

[25] Cortina J., Rottinghaus B. (2019). Vote centers and turnout by election type in Texas. Res. Politics.

[26] Manlove J. (1998). The influence of high school dropout and school disengagement on the risk of school-age pregnancy. J. Res. Adolesc..

[27] Kappeler E.M. (2015). Adolescent Health and Teen Pregnancy in the United States: A Progress Report. Public Health Rep..

[28] Yee C.W., Cunningham S.D., Ickovics J.R. (2019). Application of the social vulnerability index for identifying teen pregnancy intervention need in the United States. Matern. Child Health J..

[29] WhoLeads.Us *Confronting the Demographics of Power: America’s Sheriffs*. San Francisco, CA. June 2020. https://wholeads.us/wp-content/uploads/2020/06/reflectivedemocracy-americassheriffs-06.04.2020.pdf.

[30] Gelman A., Hill J. (2007). Data Analysis Using Regression and Multilevel/Hierarchical Models.

[31] Kirby D. (2001). Emerging answers: Research findings on programs to reduce teen pregnancy (summary). Am. J. Health Educ..

[32] Boonstra H.D. (2014). What is behind the declines in teen pregnancy rates?. Guttmacher Policy Rev..

[33] Tabi M.M. (2002). Community perspective on a model to reduce teenage pregnancy. J. Adv. Nurs..

[34] Hoffman S.D., Maynard R.A. (2008). Kids Having Kids: Economic Costs & Social Consequences of Teen Pregnancy.

[35] Hamilton B.E., Martin J.A., Ventura S.J. (2015). National vital statistics reports. National Vital Statistics Reports.

[36] Martin J.A., Hamilton B.E., Ventura S.J., Osterman M.J., Wilson E.C., Mathews T.J. (2012). Births: Final Data for 2010.

[37] Cunnington A.J. (2001). What’s so bad about teenage pregnancy?. BMJ Sex. Reprod. Health.

